# Characterization and Antioxidant Activity of Products Derived from Xylose–Bovine Casein Hydrolysate Maillard Reaction: Impact of Reaction Time

**DOI:** 10.3390/foods8070242

**Published:** 2019-07-04

**Authors:** Kun Chen, Jiajia Zhao, Xiaohan Shi, Qayum Abdul, Zhanmei Jiang

**Affiliations:** Key Laboratory of Dairy Science (Ministry of Education), College of Food, Northeast Agricultural University, Harbin 150030, China

**Keywords:** bovine casein hydrolysate, xylose, Maillard reaction product, antioxidant activity

## Abstract

The characterization and antioxidant activity on Maillard reaction products (MRPs) derived from xylose and bovine casein hydrolysate (BCH) was investigated at 100 °C and initial pH 8.0 as a function of reaction time. The pH values and free amino groups contents of xylose–BCH MRPs remarkably decreased with the reaction time up to 8 h, whereas their browning intensities significantly increased (*p* < 0.05). After 4 h of heat treatment, the fluorescence properties of xylose–BCH MRPs reached the maximum. There was a production of higher and smaller molecular substances in xylose–BCH MRPs with an increased reaction time, as analyzed by size exclusion chromatography. The 2,2-diphenyl-1-picryl-hydrazyl (DPPH) free radical scavenging capacity and ferrous reducing activity of xylose-BCH MRPs gradually increased with the reaction time extended from 0 to 8 h.

## 1. Introduction

The Maillard reaction (MR) is a reaction that occurs between carbonyl groups of reducing sugars (monosaccharides, disaccharides, or polysaccharides) and amino groups, peptides, or proteins [1], and has been recognized as a promising and effective method in protein modification to improve the antioxidant properties of proteins or peptides. The 2,2-diphenyl-1-picryl-hydrazyl (DPPH) free radical scavenging capacity of tuna backbone hydrolysate was enhanced 16-fold in the presence of tagatose after heat treatment of 55 °C for 48 h [2]. For instance, reducing the activity and DPPH free radical scavenging capacity of galactose-porcine plasma protein Maillard reaction products (MRPs) was higher than those of glucose or fructose-porcine plasma protein MRPs [3]. A variety of mechanisms have been explored to alter the antioxidant activity of Maillard reaction products, such as the splitting capability of the free radical chain [4,5], the scavenging capability of superoxide anions and hydroxyl radicals [6], metal chelation capability, and the decomposing ability of hydrogen peroxides [7]. Moreover, it has been reported that Maillard reaction products have strong antioxidant capacity due to the production of melanoidin and Amadori rearrangement products [8].

The Maillard reaction is affected by many aspects, including temperature, pH, water content, duration of heating, type of reactant, oxygen, ratio of amino acid to sugar, metals, and reaction inhibitors [9,10]. Furthermore, reaction time is the most crucial and interdependent factor that affects the reaction process and the glycation degree of the Maillard reaction [11]. Xylose can be extracted from agricultural wastes (such as corn cobs, straws, etc.), thus being a relatively inexpensive reducing pentose [12]. Due to its extensive availability, low cost, and stale physicochemical properties, it has a wide range of application in the food industry, especially for the production of xylitol and bacteria fermentation of ethanol [13]. However, xylose could also be used as a substrate for Maillard reaction to provide carbonyl groups. Furthermore, there is no evidence as to how xylose would improve the antioxidant activity of bovine casein hydrolysate (BCH) as function of the reaction time. Therefore, in this study, a xylose-BCH model system is established to elucidate the effect of the reaction time on pH, browning intensity, fluorescence property, molecular size distribution, free amino groups contents, and antioxidant activity of BCH and their possible MRPs.

## 2. Materials and Methods

### 2.1. Materials

Casein was obtained from the bovine milk using the isoelectric precipitation generated by the College of Food, Northeast Agriculture university (Harbin, Heilongjiang Province, China). Xylose was purchased from the Huishi biochemical reagent co., LTD (Shanghai, China). ASI.398 neutral protease (from *Bacillus subtilis*, 50,000, U/g), was purchased from Wuxi Enzyme Preparation Company (Wuxi, Jiangsu Province, China). OPA (O-phthaldialdehyde), bovine serum albumin (66,409 Da), ovalbumin (44,300 Da), trypsin inhibitor (20,100 Da), β-lactoglobulin (36,000 Da), lysozyme (14,300 Da), α-lactalbumin (14,147 Da), oxidized glutathione (612.63 Da), reduced glutathione (307.32 Da), L-leucine, trifluoroacetic acid (TFA), potassium ferricyanide, and 2,2-diphenyl-1-picryl-hydrazyl (DPPH) were purchased from the Sigma-Aldrich Co. (St. Louis, MO, USA).

### 2.2. Preparation of Bovine Casein Hydrolysate (BCH)

Casein was dissolved in pH 7.4 phosphate buffer (0.04 mol/L) to an initial protein concentration of 75 g/L. The enzyme concentration of AS1.398 neutral protease was fixed to 5% (*w/w* protein). The temperature during hydrolysis was kept at 45 °C in a stirred water bath. Accordingly, the pH value of the hydrolysis system was controlled at 7.0 by the continuous addition of 1 mol/L NaOH using a pH meter (Sartorius, Co., Ltd., Shanghai, China). After a 6-h hydrolysis reaction, AS1.398 neutral protease was inactivated by heating at 95 °C for 10 min. The obtained hydrolysate was centrifuged at 4000 g for 30 min, and the supernatant was ultrafiltrated through a 10-kDa molecular weight cut-off membrane under a pressure of 20 bar at 25 °C. The obtained permeates (less than 10 kDa) were lyophilized as bovine casein hydrolysate.

### 2.3. Preparation of Maillard Reaction Products (MRPs)

Bovine casein hydrolysates (3 g) and xylose (3 g) were mixed in 100 mL of distilled water. A compound of casein peptides and xylose were put in a sealed screw-top glass tube, and their initial pH was regulated to 8.0 with 4 mol/L NaOH. Sample solutions were heated at 100 °C in an oil bath equipped with a continuous stirring system, and collected after thermal treatment of 0, 1, 2, 3, 4, 5, 6, 7 and 8 h, respectively. Subsequently, sample solutions were immediately quenched in ice water and lyophilized. Heated bovine casein hydrolysates and xylose (60 mg/mL) were also conducted as control experiments. Besides, the pH value of sample solutions was measured at 25 °C.

### 2.4. Measurement of Browning Intensity

According to the techniques of Morale [5], the browning of the Maillard reaction can be monitored by the increase in the absorbance at 420 nm. Samples were diluted to a concentration of 0.3 mg/mL with distilled water.

### 2.5. Analysis of Fluorescence Properties

The excitation and emission spectrum of xylose-BCH MRPs and BCH samples were determined through a F-4500 Fluorescence Spectrometer (Hitachi. Co. Ltd., Tokyo, Japan). Samples were diluted at a protein concentration of 0.3 mg/mL. The slits were at 5 nm with the scan rate of 120 nm/min. At the excitation wavelength of 340 nm, emission spectra were carried out from 370 to 600 nm. Accordingly, at the emission wavelength of 425 nm, excitation spectra were completed from 300 to 400 nm.

### 2.6. Determination of Free Amino Groups Content

The free amine groups of xylose BCH MRPs and BCH samples were calibrated by OPA methods [13]. In short, a 30-mL sample was mixed up with 1 mL of OPA reagent. A blank reading was determined by using distilled water. The change in free amino groups contents was finally showed as relative concentrations (%) compared to that of unheated samples.

### 2.7. The Molecular Size Distribution of MRPs

The size exclusion chromatography of xylose-BCH MRPs and BCH samples were performed by using a TSK gel G 2000 SW (600 × 7.5 mm,10 μm, Tosoh Corporation, Tokyo, Japan) equipped with TSK guard column (75 × 7.5 mm, 10 μm). The eluent was 30% acetonitrile solution containing 0.1% TFA distilled water and the flow rate is 0.5 mL/min. First, 20-μL samples (2.5 g/L) were injected, and their absorbances were measured at 214 nm. Bovine serum albumin (66,409 Da), ovalbumin (44,300 Da), trypsin inhibitor (20,100 Da), β-lactoglobulin (36,000 Da), lysozyme (14,300 Da), α-lactalbumin (14,147 Da), oxidized glutathione (612.63 Da), and reduced glutathione (307.32 Da) were adopted as molecular weight (MW).

$$\mathrm{Log}\mathrm{MW}=-0.1082x+7.573,\mathrm{with}\;\mathrm{elution}\;\mathrm{time}\;\mathrm{expressed}\;\mathrm{in}\;\mathrm{minutes},\;R^{2}=0.9878$$

### 2.8. Measurement of DPPH Free Radical Scavenging Capacity

The 2,2-diphenyl-1-picryl-hydrazyl (DPPH) free radical scavenging capacity of xylose-BCH MRPs and BCH samples was analyzed using the method of Saiga et al. [14]. The sample (160 μL) was dissolved in 1 mL of a DPPH solution, which was daily prepared at 0.1 mol/L in ethanol. With 95% ethanol as the reference solution, after the mixture was centrifuged at 4000 g for 10 min, its absorbance was observed at 517 nm. The percentage of DPPH free radical scavenging capacity was calculated as follows:

(1) $$\mathrm{DPPH}\;\mathrm{free}\;\mathrm{radical}\;\mathrm{scavenging}\;\mathrm{capacity}\%=\left(1-\frac{A-A_{i}}{A_{j}}\right)\times 100\%$$

where *A* is the absorbance of samples and DPPH ethanol, *A_i_* is absorbance of samples and 95% ethanol, and *A_j_* is the absorbance of DPPH ethanol without samples.

### 2.9. Measurement of Ferrous Reducing Activity

The ferrous reducing activity of xylose-BCH MRPs and BCH was observed on the basis of the method of Zhu et al. [15]. First, a 0.1-mL sample was diluted 12-fold and mixed with 0.5 mL of 0.2 mol/L Na_3_PO_4_ (pH 6.6) and 0.5 mL of 1% potassium ferricyanide. After centrifugation at 25 °C for 10 min, 0.5 mL of supernatant was withdrawn and mixed with 0.5 mL of distilled water and 0.2 mL of 0.1% (*w*/*v*) ferric chloride solution. Subsequently, this mixture was maintained at 50 °C for 20 min; then, 10% TFA (0.5 mL) was added. Furthermore, after a further reaction for 10 min at room temperature, its absorbance was recorded at 700 nm.

### 2.10. Statistical Analysis

Analyses including the measurement of pH, browning intensity, molecular size distribution of MRPs, fluorescence property, free amino groups contents, DPPH free radical scavenging capacity, and ferrous reducing activity were run in triplicate. Size exclusion chromatography measurements were carried out in duplicate. All the data are expressed as mean ± standard deviation (SD). By using Duncan’s multiple range tests by the SPSS system, a difference of means were identified (SPSS 19.0 for Mac OS X, SPSS Inc., Chicago, IL, USA).

## 3. Results and Discussion

### 3.1. Effect of Reaction Time on pH of Xylose-BCH MRPs

**Figure A1:**
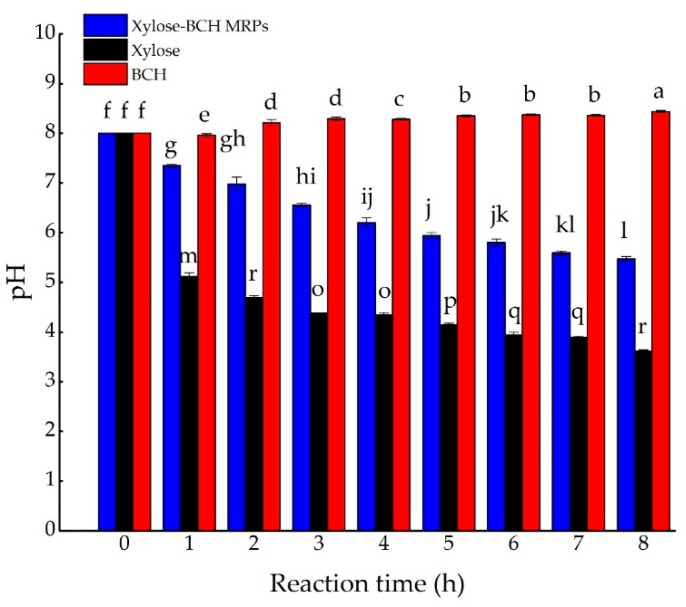
Effect of Maillard reaction time on the pH of xylose–bovine casein hydrolysate (BCH) MRPs at 100 °C. The standard deviation is showed by error bars. Data points with diverse letters are significantly different (*p* < 0.05).

The effect of the Maillard reaction time on the pH of xylose-BCH MRPs was shown in Figure A1. There was a significant difference in the pH changes between BCH and xylose-BCH MRPs. The pH of xylose-BCH MRPs was remarkably lower than that of heated BCH with the Maillard reaction time increasing from 0 to 8 h (*p* < 0.05). The pH of heated BCH slightly increased with the Maillard reaction time extended from 0 to 8 h, potentially on account of the thermal protein degradation [16]. However, the pH of xylose-BCH MRPs and heated xylose greatly decreased after the heat treatment of 8 h (*p* < 0.05). The pH of heated xylose was the minimum compared to that of xylose-BCH MRPs and BCH during heat treatment of 8 h, which was probably due to its sugar degradation being much faster under alkaline pH conditions [17]. Accordingly, the initial pH of xylose-BCH MRPs dropped from 8.0 to 5.47 after 8 h of thermal treatment. The pH of casein-glucose MRPs also decreased at 102 °C with the reaction time extended from 0 to 130 min [18]. The decrease in the pH was potentially owing to the production of organic acids, including formic acid and acetic acid derived from intermediate MRPs [1]. Additionally, Rufianhenares et al. [19] reported that 1-deoxyglucosone and 3-deoxyglucosone produced from monosaccharide degradation products further formed formic and acetic acids. Meanwhile, it was also found that the pH value of the galactose–glycine model system decreased as the sugar concentration increased at 60 °C, 75 °C, and 90 °C [1]. Besides, Liu et al. [20] proposed that the decrease in pH is also related to the formation of reductones and melanoidins, leading to increased reducing power and radical scavenging capacity.

### 3.2. Effect of Reaction Time on Browning Intensity of Xylose-BCH MRPs

**Figure A2:**
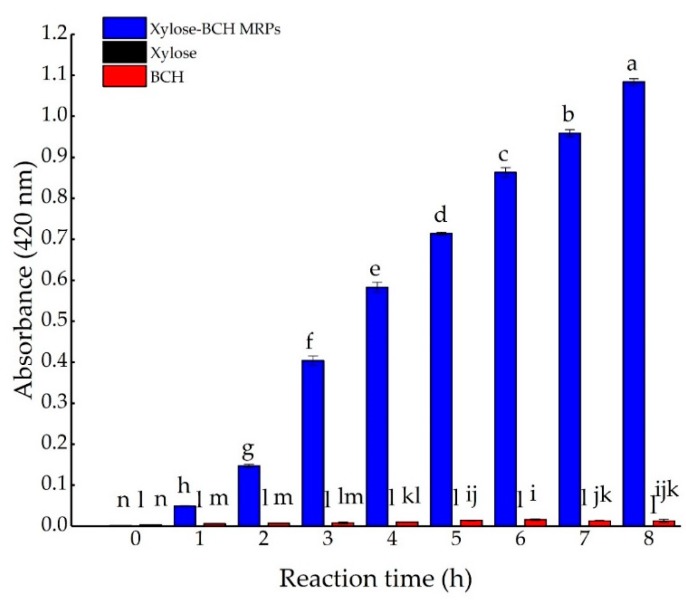
Effect of Maillard reaction time on browning intensity of xylose–BCH Maillard reaction products (MRPs) at pH 8.0 and 100 °C. The standard deviation is showed by error bars. Data points with diverse letters are significantly different (*p* < 0.05).

The occurrence of a Maillard reaction is indicated by the development of a brown color, and usually monitored by absorbance increase at 420 nm [5]. The advanced reaction stage involves condensation, leading to the formation of oligomers and polymers of high molecular weight, and brown color as melanoidins [21,22]. The effect of reaction time on the browning intensity of xylose-BCH MRPs was shown in Figure A2. The browning intensity of xylose-BCH MRPs was higher than that of xylose or BCH, and significantly increased with a reaction time up to 8 h (*p* < 0.05). A similar result was found for the browning of porcine plasma proteins as a consequence of the increasing time [3]. Moreover, the colored components of nonenzymatic browning might contain the low or high molecular weight products and melanoidins. Under less acidic conditions, the reactive cyclic compounds such as hydroxy methyl furfural were polymerized to a dark-colored insoluble substance containing nitrogen atoms [23]. In comparison, the browning intensity of xylose and heated BCH did not remarkably change during the thermal treatment of 8 h (*p* > 0.05). It was also confirmed that the browning of the sugar–amino acid mixture was derived from MRPs without sugar caramelization [24].

### 3.3. Effect of Reaction Time on Fluorescence Properties of Xylose-BCH MRPs

**Figure A3:**
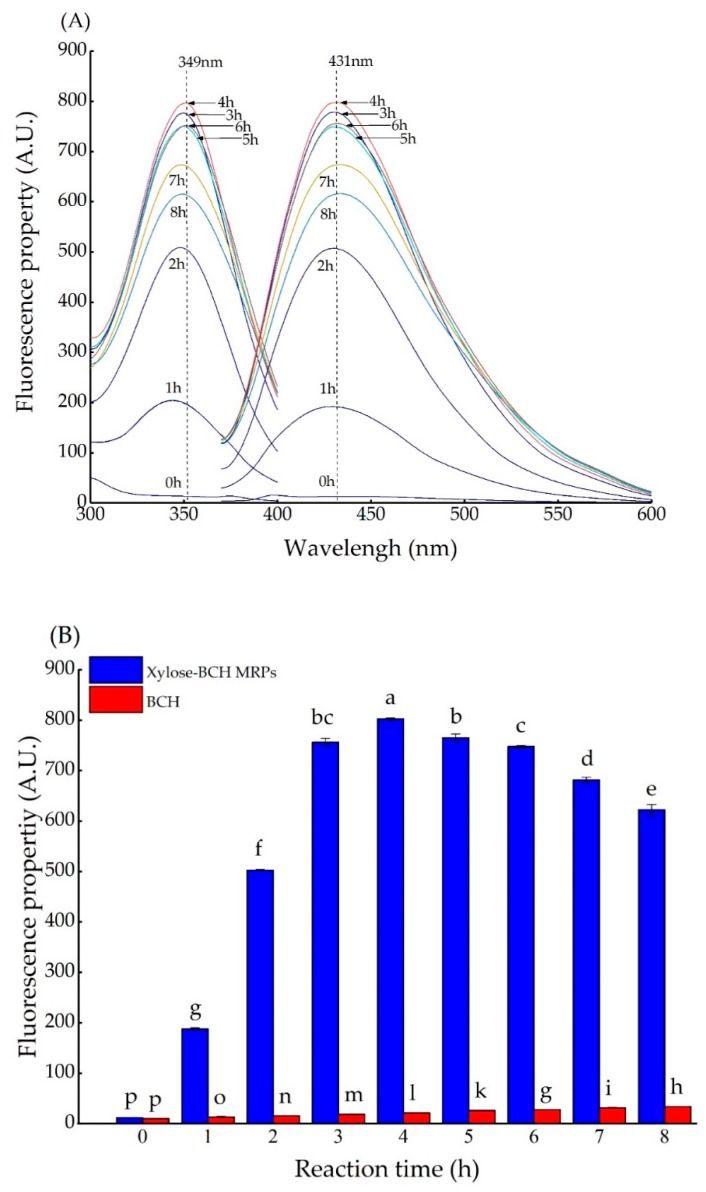
Effect of Maillard reaction time on the fluorescence spectrum (**A**), and fluorescence property (**B**) of xylose–BCH MRPs. In (**B**), a comparison with BCH was reported, and data points with diverse letters are significantly different (*p* < 0.05).

The fluorescent compounds are precursors that form brown pigments during the Maillard reaction [25]. Generally, there was a different trend regarding the fluorescence development between BCH and xylose-BCH MRPs. Fluorescence spectral analysis was previously used to characterize the Maillard reaction [26]. The effect of reaction time on the fluorescence spectrum and fluorescence property of xylose-BCH MRPs is shown in Figure A3A,B, respectively. The optimal excitation and emission wavelength of xylose-BCH MRPs was detected at 349 ± 1 and 431 ± 1 nm, respectively. The fluorescence property of xylose-BCH MRPs initially increased, and afterwards decreased with the reaction time extended up (*p* < 0.05). This indicated that the reaction time could remarkably affect the patterns of fluorescent formation in xylose-BCH MRPs. It is also reported that the development of fluorescent compounds of α-lactalbumin-ribose and β-lactoglobulin-ribose had a similar trend with reaction time [27]. However, the fluorescence property of ribose-casein MRPs increased as a function of reaction time [28].

After heat treatment for 4 h, the fluorescence property of xylose-BCH MRPs reached the maximum, and its fluorescence property was approximately 66-fold higher than that of unheated xylose-BCH MRPs. Furthermore, the fluorescence properties of hydrolyzed β-lactoglobulin and heated glucose was only approximately 1% and 2% of the fluorescence property in glucose-β-lactoglobulin MRPs after 18 h of heat treatment at 100 °C [29]. Likewise, Saeki et al. [30] demonstrated that the ribose-casein MRPs displayed a 10-fold greater fluorescence property than glucose-casein and fructose-casein MRPs, which reached a maximum and then plateaued after four days of heating. These results indicated that the long heat treatment could influence the fluorescent development patterns of MRPs.

### 3.4. Effects of Reaction Time on the Loss of Free Amino Groups Content of Xylose-BCH MRPs

The carbonyl groups of reducing sugars react with ε-NH_2_ groups of amino acid and terminal α-amino groups of peptides at an early stage of the MR [31]. Therefore, the loss of free amino groups content determined with the OPA method could be an indicator in the reaction degree of the Maillard reaction.

**Figure A4:**
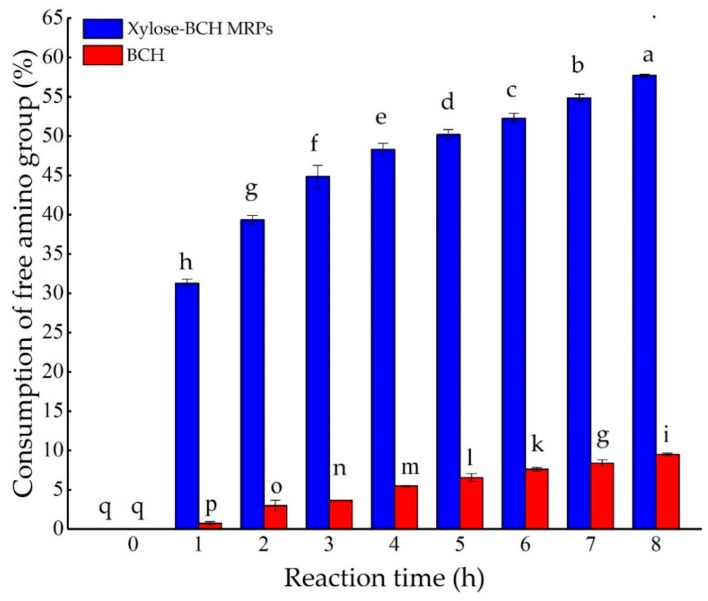
Effect of reaction time on the loss of free amino groups content of xylose–BCH MRPs and BCH. The standard deviation is showed by error bars. Data points with diverse letters are significantly different (*p* < 0.05).

The effect of reaction time on the loss of free amino groups in heated BCH and xylose-BCH MRPs was shown in Figure A4. The loss of free amino groups in xylose-BCH MRPs was higher than that in BCH (*p* < 0.05). The loss of free amino groups in heated BCH and xylose-BCH MRPs continuously decreased during the thermal treatment of 8 h (*p* < 0.05). For example, the loss of free amino groups of BCH and xylose-BCH MRPs was 9.48% and 57.71%, respectively. This result was in accordance with those of Dong et al. [29], which demonstrated that the disappearance of free amino groups also occurred in the Maillard reaction between hydrolyzed β-lactoglobulin and glucose at 90 °C for 18 h. Zeng et al. [2] also proved that the disappearance of free amino groups also occurred in the Maillard reaction between four reducing sugars (fructose, psicose, sorbose, and tagatose) and a tuna backbone hydrolysate of up to 48 h at 55 °C. Differently, the free amino groups of the psicose-lysine and fructose-lysine systems both decreased sharply and were subsequently followed by a steady state during the thermal treatment [32]. It was further reported that the consumption of amino groups in bovine serum albumin (BSA) glucose was higher than those of heated BSA [33].

### 3.5. Effect of Reaction Time on Molecular Size of Xylose-BCH MRPs

**Figure A5:**
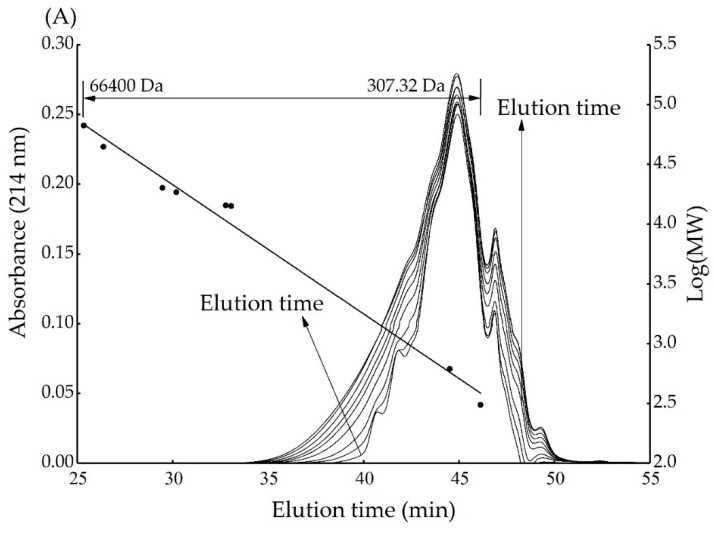

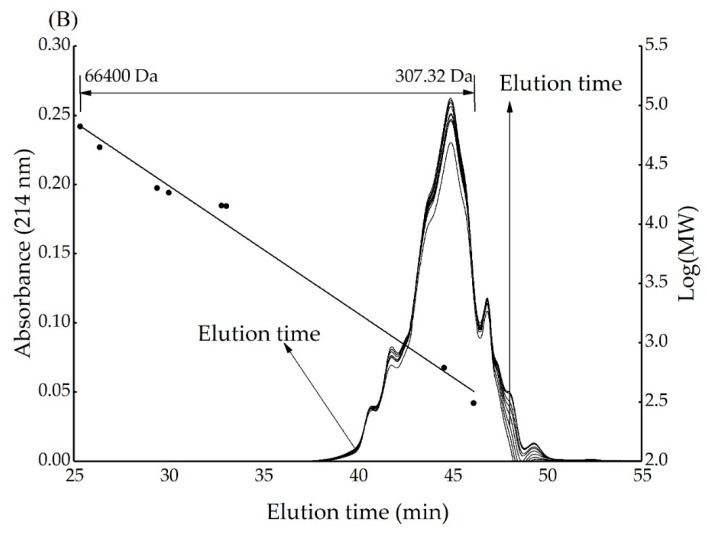
Effect of reaction time on molecular size of xylose–BCH MRPs (**A**) and BCH (**B**).

The effect of reaction time on the molecular size of xylose-BCH MRPs and heated BCH was shown in Figure A5A,B. There are similar shapes in the chromatogram with the same peaks showing increasing areas at increasing reaction times. This observation indicated that a large amount of MRPs with different molecular weight were generated.

The main molecular weight of unheated xylose-BCH MRPs was about 2388 Da. Following the 8 h treatment, it becomes significantly higher (7551 Da), thus indicating that a molecular rearrangement of peptide bonds takes place. This finding was already reported in the literature [34], and was also found concerning the covalent cross-linking between BCH and galactose [35] as well as the aggregation found between α-lactalbumin or β-lactoglobulin and ribose during heat treatment [27].

### 3.6. Effect of Reaction Time on Antioxidant Activity of Xylose-BCH MRPs

#### 3.6.1. DPPH Free Radical Scavenging Capacity

**Figure A6:**
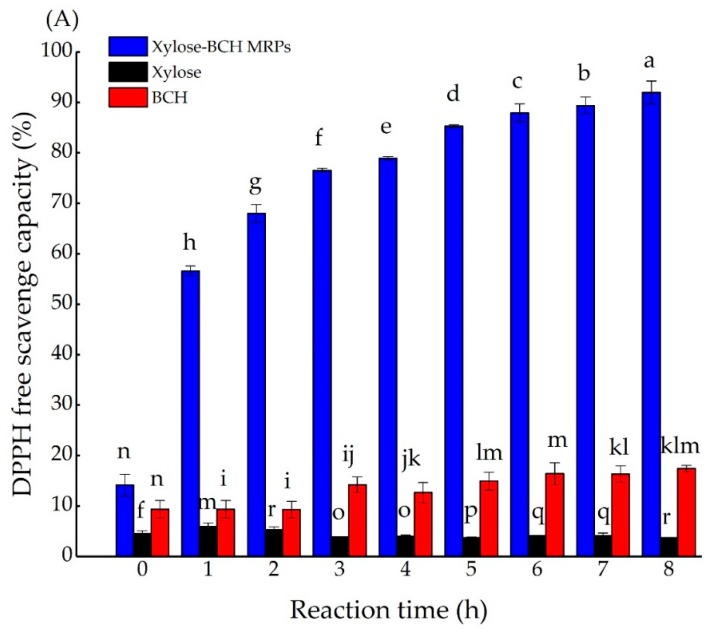

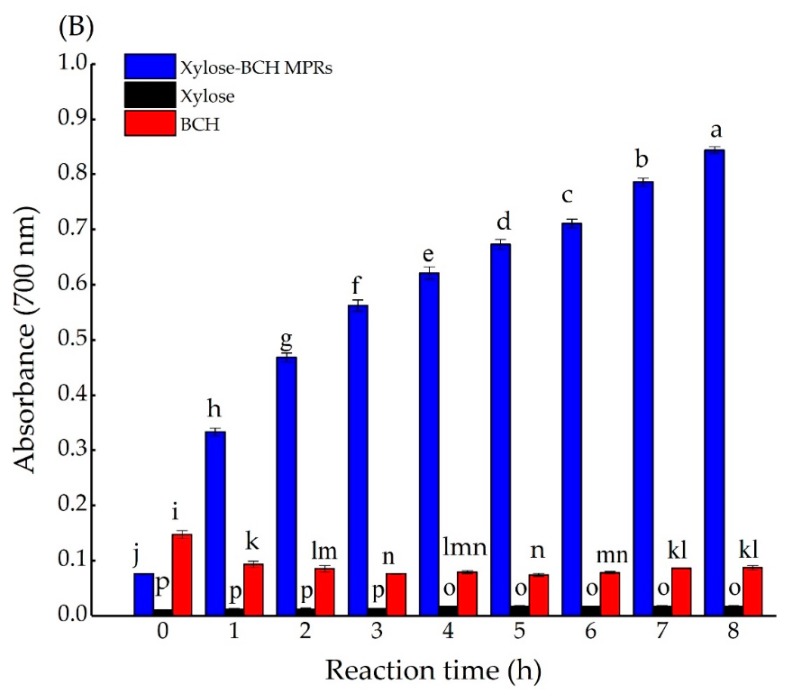
Effects of the reaction time on 2,2-diphenyl-1-picryl-hydrazyl (DPPH) free radical scavenging capacity (**A**) and on ferrous reducing activity (**B**) of xylose–BCH MRPs, BCH, and xylose samples. Effects of reaction time on absorbance at 700 nm of xylose–BCH MRPs, BCH, and xylose (**B**). The standard deviation is showed by error bars. Data points with diverse letters are significantly different (*p* < 0.05).

During the Maillard reaction, final brown polymers or intermediates can function as hydrogen donors and can be beneficial to scavenge DPPH free radical scavenging capacity [3,36]. The DPPH free radical scavenging capacity of xylose-BCH MRPs and BCH significantly increased during the whole reaction time (*p* < 0.05), as shown in Figure A6. These results were similar to those reported in the past [27]. The DPPH free radical scavenging capacity can be shown by MRPs from sugar amino groups, sugar peptide and sugar protein model systems [27,32]. Furthermore, Chen et al. proved that the size of the peptides could affect the antioxidant ability [37]. The DPPH free radical scavenging capacity of xylose-BCH MRPs was stronger than that of BCH. Accordingly, the DPPH free radical scavenging capacity of MRPs was 22-fold higher than that of BCH after 8 h of thermal treatment. The DPPH free radical scavenging capacity of xylose-BCH increased from 14.12% to 91.98%, corresponding to 1 h and 8 h, respectively (*p* < 0.05), which is part of the reason that the sugar caramelization can cause the destruction of DPPH radical activity [3]. The DPPH free radical scavenging capacity of MRPs had similar activities with (*p* < 0.05) increased from 67.95% to 78.91% with a heating time of 3 to 5 h. Besides, Benjakul et al. showed that the DPPH free radical scavenging capacity of MRPs derived from galactose and fructose increased as the heating time extended [3]. Similarly, the DPPH free radical scavenging capacity of hydrolysate from casein and fish was improved by 20–30% when reacted with glucose [38]. Additionally, grass carp protein–xylose MRPs showed antioxidant activity in virtue of the production of large quantities of pyrazines, furans N-heterocyclic compounds, and ketones [39]. Furthermore, it was also proved that the antioxidative effects of scallop female gonad hydrolysates–ribose MRPs might be due to the intermediate chemicals (including furans, reductones, and pyrazines), and melanoidin manufactured during heating [40].

#### 3.6.2. Ferrous Reducing Activity

Ferrous reducing activity is also an assay method of evaluating antioxidant activity [35]. The hydroxyl and pyrrole groups of MRPs are important ways to contribute to the reducing activity [41,42]. The effects of reaction time on the ferrous reducing activity of xylose–BCH MRPs, BCH, and xylose are shown in Figure A6B. There was a significant difference in the ferrous reducing activity between BCH and xylose–BCH MRPs. After the thermal treatment of 8 h, the ferrous reducing activity of xylose–BCH MRPs increased significantly as the reaction time increased (*p* < 0.05), being attributed to formed colored melanoidins from aldol condensation and aldehyde–amine polymerization in the final stage [43]. MRPs have been reported to quench hydrophilic free radicals more efficiently than hydrophobic free radicals [30]. Furthermore, the pyrrole and hydroxyl groups of MRPs, through their redox potential of transferring electrons, have a vital role in the process of ferrous reduction. This was in line with the opinions of Shazly et al. [44], who reported that there could be a higher reducing power for glucose–glycine MRPs as the heating time increased. The ferrous reducing activity of a porcine hemoglobin hydrolysate–sugar model system was about four times higher than that of individual hydrolysate [3]. Actually, the results showed that there could be a positive correlation between the ferrous reducing activity or DPPH free radical scavenging activity and browning intensity.

## 4. Conclusions

This study represents the first evaluation of the impact of reaction time on the antioxidant activity and physicochemical properties of a xylose–BCH system. Up to 8 h of reaction time, the pH values and free amino groups content of xylose–BCH MRPs remarkably decreased. After heat treatment of 4 h, the fluorescence property of xylose–BCH MRPs reached the maximum value. The molecular weight distribution range gradually increased, and more polymeric substances were formed. The DPPH free radical scavenging capacity and ferrous reducing activity of xylose–BCH MRPs gradually increased. Therefore, this study showed an effective method for the Maillard reaction to ameliorate the antioxidant activity of bovine casein hydrolysate.
